# The Protective Role of Neurogenetic Components in Reducing Stress-Related Effects during Spaceflights: Evidence from the Age-Related Positive Memory Approach

**DOI:** 10.3390/life12081176

**Published:** 2022-08-02

**Authors:** Nicola Mammarella, Matteo Gatti, Irene Ceccato, Adolfo Di Crosta, Alberto Di Domenico, Rocco Palumbo

**Affiliations:** 1 Department of Psychological Sciences, Health and Territory, University G. d’Annunzio of Chieti-Pescara, 66100 Chieti, Italy; matteo.gatti@unich.it (M.G.); adolfo.dicrosta@unich.it (A.D.C.); alberto.didomenico@unich.it (A.D.D.); rocco.palumbo@unich.it (R.P.); 2 Department of Neuroscience, Imaging and Clinical Sciences, University G. d’Annunzio of Chieti-Pescara, 66100 Chieti, Italy; irene.ceccato@unich.it

**Keywords:** positive emotions, positivity effect, spaceflight, long-term space missions, ADRA2B, COMT, CB1, 5HTTLPR, TOMM40

## Abstract

Fighting stress-related effects during spaceflight is crucial for a successful mission. Emotional, motivational, and cognitive mechanisms have already been shown to be involved in the decrease of negative emotions. However, emerging evidence is pointing to a neurogenetic profile that may render some individuals more prone than others to focusing on positive information in memory and increasing affective health. The relevance for adaptation to the space environment and the interaction with other stressors such as ionizing radiations is discussed. In particular, to clarify this approach better, we will draw from the psychology and aging literature data. Subsequently, we report on studies on candidate genes for sensitivity to positive memories. We review work on the following candidate genes that may be crucial in adaptation mechanisms: ADRA2B, COMT, 5HTTLPR, CB1, and TOMM40. The final aim is to show how the study of genetics and cell biology of positive memory can help us to reveal the underlying bottom-up pathways to also increasing positive effects during a space mission.

## 1. Introduction

The psychology and aging literature can inform us about the role of positive memory in coping with stress-related responses during a space mission. In fact, it is known that there are many spaceflight parallels to aging effects on the human body and mind, as the impact of microgravity (MG) resembles a pattern of physiological and psychological age-related changes typically observed on Earth, including a series of effects on the immune system, bones, muscles, balance, and hand-eye coordination [1]. Positive emotions strongly influence mental health in later life, as they impact on the healthy habits, physiological systems, stress exposure, and coping mechanisms of older adults [2]. In particular, positive emotions are known to have an antistress effect [3], as they can change the intensity and severity of stress reactions, physiological indices (e.g., heart rate variability and blood pressure), and as a result, the individual susceptibility to disease [4].

The behavioral literature on healthy aging is rich in studies indicating a cognitive advantage for the processing of positive information (see [5] for a review) and pointing to the “positivity effect” [6] as a crucial controlled-based mechanism that allows older adults to adapt to age-related changes. Specifically, when older participants are presented with valenced stimuli and are asked to remember them across different memory tasks (e.g., with verbal or pictorial stimuli that are positively charged, as in [7]), positive stimuli usually result in greater memory performance compared with both negative and neutral ones [8]. This successful motivational strategy is used to increase the everyday positive affect and, consequently, to reduce the likelihood of experiencing negative emotions. In particular, healthy older adults tend to use positive memories to cope with the thoughts that their time left is limited. Neuroimaging research also identified a specific brain pattern of activation during positivity effects in memory, with higher levels of activation in the prefrontal cortex (PFC) of healthy older adults for positive stimuli compared with younger adults. Furthermore, an age by valence interaction in amygdala activation revealed that only older persons had greater amygdala activity in reaction to positive rather than negative images. Finally, during the processing of emotional content, there is an increased functional connection between the PFC and the amygdala, which may indicate older individuals’ attention shifting to positive information.

The relevance of positive emotions and affective health in healthy aging is capturing the attention of many researchers from the space sciences domain as well [9,10,11]. In fact, given the above mentioned parallel between aging and astronauts’ health, and in line with the broaden-and-build theory of positive emotions [4], pleasant emotions may also mediate successful adaptation to interplanetary missions, such as by magnifying individual stress resilience traits.

Our interest in this particular cognitive bias stems from the fact that positivity effects in memory can be viewed as a robust coping strategy that astronauts may also use to fight space-related stressors during a mission. This may happen for two reasons. First, even in situations where there are fewer cognitive resources available, such as under MG conditions, the positivity effect may occur. Second, the evidence outlined above suggests that additional mechanisms aside from behavioral ones may be at work (as the neuroimaging data also seem to suggest); that is, the biological foundations (such as genes) may be considered fundamental to the understanding of individuals’ general sensitivity to positive information in memory. This bottom-up explanation of the effect allows drawing a specific profile that may render some individuals more prone than others to focusing on positivity-laden information.

While some authors have already emphasized the importance of emotional health in long-term missions [9], here, we aim to outline the genetic profile related to the positivity effect in memory. In the following paragraph, we review the evidence for a series of genes that, alone or in combination, may modulate the occurrence of positivity effects in older adults’ memory and help identify a neurogenetic profile for individuals who are better able to adapt to stressful situations.

## 2. Materials and Methods

The Preferred Reporting Items for Systematic Reviews and MetaAnalyses (PRISMA) statement was used to perform and recode this review [12].

### 2.1. Eligibility Criteria

The inclusion criteria were the relationship between effects (emotions, moods, stress responses, and coping strategies) and gene candidates, relationships between adaptation to extreme environments and biological underpinning, emotional countermeasures used in space missions or publications proposing countermeasures for space missions, and articles written in English.

The exclusion criteria were irrelevant topics, studies with insufficient data (e.g., research site, study time, and sample size), interventional studies, case reports, editorial comments, and letters to editor.

### 2.2. Information Sources

A systematic electronic search was undertaken in the following databases between March 2022 and May 2022 with no time limits: PubMed, Scopus, PsychINFO, and Google Scholar. Where applicable, MeSH and Emtree terminology were utilized. A handsearch of relevant studies was also carried out. The Rayyan online program [13] was used to import and categorize all of the references.

### 2.3. Search Strategy

Computer searches of the PubMed, PsycINFO, Scopus, and Google Scholar databases were used to perform this review. These searches were run until 31 May 2022.

There were two stages to the article selection process. There was no time limit, no search filters, and no constraints. The first search included the keywords “ADRA2B”, “COMT”, “CB1”, “5HTTLPR”, and “TOMM40”. Then, we searched for some combinations of keywords between the above-mentioned candidate genes and “positive emotions”, “spaceflight”, “stress-related responses”, “spaceflight”, “space exploration”, “long-term space missions”, and “adaptation to extreme environments”. The articles were identified and then listed in an Excel table. Titles and abstracts unrelated to the topic were then excluded, as well as duplicate records. As a result, we read the entire content during the eligibility verification step. All items selected in the previous phases were examined for quality evaluation in the final step, and low-quality studies (i.e., insufficient data provided) were excluded. Zotero software version 6.0.6 was used to handle all references [14].

### 2.4. Data Collection

Following the application of the search strategy, a total of 201 works were identified. Of those, 175 publications were screened after the duplicates were removed. Studies were first included for their titles and abstracts’ relevance to the research question. Following that, the screening process resulted in the retrieval of 100 works. The full text of the remaining articles was obtained and evaluated for eligibility. Seventy-three reports were reviewed for eligibility and then ranked according to the inclusion criteria. Finally, we included in the present review 64 publications.

**Figure 1:**
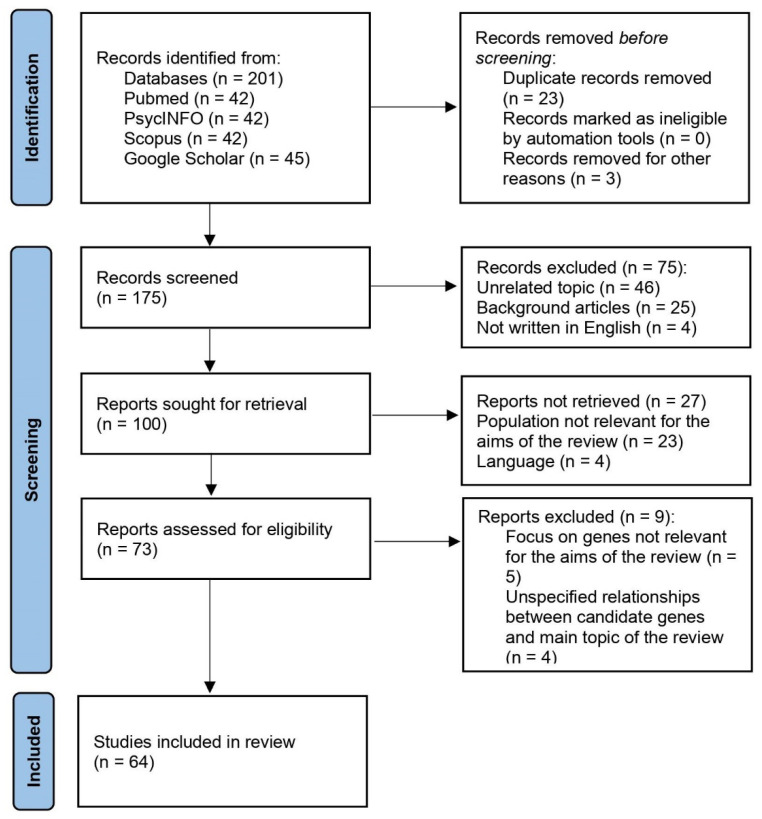
PRISMA flow diagram of the selection process.

Figure 1, the PRISMA flow diagram, depicts the selection process as well as the inclusion decision [12].

## 3. A Neurogenetic Profile for Sensitivity to Positive Memories: ADRA2B, COMT, 5HTTLPR, CB1, and TOMM40

Monoamines (e.g., noradrenaline (NA), dopamine (DA), and serotonin (5-HT)) are necessary in emotion regulation processes. NA, DA, and 5-HT genes in particular are hypothesized to modulate emotion and memory interactions via regulating a wide variety of cognitive and emotional functions [15]. Below, we will discuss some of the key polymorphisms involved in positive emotions and adaptation [16]:

(i) A deletion variation of the ADRA2B gene, which affects adrenergic receptors’ activation;
(ii) The Val/Met single-nucleotide polymorphism in the COMT gene, which affects dopamine metabolism in the prefrontal cortex;
(iii) A short allele of the 5HTTLPR serotonin transporter gene;
(iv) The endocannabinoid system, which involves cannabinoid receptors type 1 (CB1);
(v) The translocase of outer mitochondria membrane 40 gene (TOMM40).

Previous research has found that these polymorphisms can influence information processing in brain areas involved in emotional memories [15] (e.g., the hippocampus, amygdala, and PFC cortex) and play an important role in emotion-cognition interactions [16,17]. The above-mentioned polymorphisms will be analyzed in relation to the aging literature. Here, we want to draw insights on the adaptation mechanisms in long-term space missions from the way individuals cope with negative emotions later in life. To this aim, we will investigate genes related to positive emotions in successful aging as effective adaptation mechanisms for later life challenges. The ultimate goal is to delineate a genetic profile that may result in a better ability to adapt to difficult environmental circumstances, such as those that astronauts may encounter during interplanetary space missions.

### 3.1. ADRA2B

Age-related changes in emotional memory may be linked to genetic polymorphisms associated with noradrenergic signaling [17], whose secretion is regulated in the amygdala. In a recent study, a functional deletion polymorphism in the α-2B adrenoceptor gene (ADRA2B) was associated with emotional memory [18]. Through presynaptic regulation of NA release, the ADRA2B deletion lowers receptor functioning while increasing central noradrenergic transmission. In terms of positive memory, deletion carriers have been found to have better recall for emotional information due to an increase in noradrenergic neurotransmission induced by emotional arousal [15]. Deletion carriers, moreover, showed an overall arousal enhancement effect in long-term memory tasks [18]. According to fMRI findings, deletion carriers have increased neural activity in the amygdala during the encoding of emotional images [19]. Noradrenergic system activity increases in older adults both peripherally and in the central nervous system compared with younger adults [16]. In healthy aging, the augmented noradrenergic activity could explain a stronger “chronic” focus on emotion. Furthermore, given recent findings linking NA to cognitive-affective flexibility and cognitive reserve [20], it is plausible that NA is strongly involved in emotion regulation mechanisms, particularly the positivity effect in memory. In fact, those older adults with finer cognitive processes are more effective in applying emotional techniques to promote well-being (e.g., more conceptually based elaboration of stimuli toward the positive pole). For example, studies suggest that deletion carriers are more frequent in healthy aging and that ADRA2B polymorphism can modify the valence of unpleasant memories in older persons. The ADRA2B gene in particular moderates traumatic memories in a sample of older people, shifting the valence of memories from negative to positive [18].

The ADRA2B deletion variation was found to be strongly related to enhanced perceptual and cognitive task performance for emotional stimuli in a recent meta-analysis of 16 published trials involving 2752 individuals [21]. When non-emotional content was employed, however, this genetic influence did not influence the overall task performance. The implications of the ADRA2B deletion variant on task performance may manifest as and rely on different amygdala brain activity in carriers and non-carriers [22,23]. Finally, a new interesting association between a single-nucleotide polymorphism (rs2400707) within the promoter region of the ADRA2B gene and PTSD symptoms in interaction with childhood trauma has been discovered (*p* = 1.02 × 10^−5^). The rs2400707 polymorphism has been linked to adrenergic system function, and more specifically, the A allele has been linked to a major resilience to childhood adversity [24].

### 3.2. COMT

The enzyme catechol-O-methyltransferase (COMT) catabolizes dopamine (DA) and is located mostly in the prefrontal cortex (PFC) and temporal brain areas. The most investigated COMT polymorphism is Val108/158Met, which has been found to alter prefrontal cortex (PFC) activity during both emotion processing and executive tasks [25]. The COMT gene is a promising candidate for explaining age-related changes within the control-based theory of the positive effect in memory. Met homozygotes in particular have increased DA levels compared with Val homozygotes, resulting in worse emotion regulation abilities. For example, COMT Met allele carriers exhibit greater activity in the amygdala and prefrontal areas when presented with negative stimuli [26]. A meta-analysis [25] found that COMT had a strong influence on prefrontal activity, with Val carriers doing worse in cognition tasks and Met carriers performing worse in emotion-related tasks. COMT could have a pleiotropic effect on motivated cognition (more sensitive to cognitive-based vs. emotion-based motivational goals). In terms of aging, Met carriers outperform Val carriers in WM and executive functions tasks [27,28]. Older people with the Met genotype exhibit greater amounts of extracellular dopamine and, as a result, have better performance in WM tasks (which depend on PFC dopamine). The Val genotype, on the other hand, is linked to lower levels of extracellular dopamine and worse WM performance [27]. This might indicate that the met158 allele is pivotal for WM and attention-related tasks, and the val158 allele is advantageous in the processing of negative stimuli [29]. At first sight, this evidence appears to contradict Mather and Knight’s theory [29,30], which claims that cognitive control is critical in modifying the direction of emotional biases in memory. In sum, individuals with lower cognitive performance (Val) are better at emotion regulation, while those with better cognitive performance (Met) are more prone to emotion dysregulation. However, a recent piece of research [31] looked at WM performance under different degrees of cognitive load (i.e., increasing task difficulty) and found no genotype-by-load interactions or main effects of the COMT genotype on accuracy. Although the COMT genotype may affect PFC activity, these effects may not be directly connected to the levels of control processes required by the task. As a result, the ideal degree of cognitive control might be linked to reduced DA levels, which would allow a better focus on positive information. We hypothesized that Val carriers might be more able to compensate for low DA levels and that this reaction could explain their improved emotion regulation abilities. During affective valence evaluation, Met carriers, on the other hand, are less able to express feelings and exhibit lower brain activity in the posterior cingulate gyrus and precuneus [32]. From a clinical point of view, the Val158Met polymorphism has been linked to worse memory performance (both immediate and delayed) and worse executive functions in patients with bipolar disorder (BD), while no significant link was found between the COMT genotype and cognitive performances in healthy subjects. Other studies on BD patients found a relationship between the val158Met polymorphism and deficits in spatial working memory [33] and in emotion recognition and regulation [34].

### 3.3. HTTLPR

Emotion processing is also affected by a functional polymorphism in the regulatory area of the serotonin transporter gene-linked polymorphic region (5HTTLPR). On chromosome 17q11.1–q12.2, this polymorphism is a 44-bp insertion (identified as the “l” allele) or deletion (designated as the “s” allele) in the transcriptional regulatory region. Long–long (l/l), short–long (s/l), and short–short (s/s) genotypes for the 5HTTLPR have been identified. S-allele carriers in particular have poorer transcriptional efficiency and reduced serotonin transporter activity, making them more vulnerable to valence effects. Defrancesco et al. [34] found that while homozygous l-carriers recognize positive emotions better and sooner, s-allele carriers recognize negative emotions instead. In a sample of female s-allele carriers for instance, there was evidence of reduced left fusiform gyrus activity to positive stimuli [35]. When compared with l-carriers, those with the s-allele show a higher emotional response to negative stimuli and a lower neurological response to positive stimuli [36]. A recent work [36] considered the putative differences in emotion regulation abilities and, in particular, the ability to suppress physiological responses to aversive visual stimuli in relation to the 5-HTTLPR s-allele polymorphism. Ninety subjects (n = 75 female; mean age = 24.53 years; SD = 9.49) took part in a within-subject experiment in which they first watched 60 affective stimuli (negatively valenced = 50, neutral = 10, with all retrieved both from the IAPS [37] and from the web) and rated their valence and arousal on a SAM scale [38]. The subjects were then told to suppress any sensations they might have had as they watched the images. In sum, each participant first saw the stimuli without any instructions (first condition) before being told to suppress any emotions before the second cycle (second condition). The word “suppress” appeared on the screen during the inter-stimulus interval in the second condition. Electrodermal activity (EDA) was recorded, and some questionnaires were administered, including the SVF 120 [39], which assesses routine coping strategies (e.g., the need for social support, distraction, avoidance, and aggression). The results showed that the 5-HTTLPR genotype influenced the skin conductance response (SCR) to emotional stimuli in the second condition but not the subjective picture judgments in the first condition. Specifically, the SCR to aversive images was lower in s-allele carriers when asked to suppress emotion, while the LL-genotype had lower levels of SCR at the baseline. Hence, the 5-HTTLPR genotype might affect the extent to which people experience emotional stimuli: s-allele carriers have an equivalent SCR to the LL-genotype when they obtain supportive guidance on how to cope with adversity. LL-genotypes, on the other hand, do not appear to need such guidance; instead, they tend to react less to unpleasant stimuli than s-allele carriers, implying that they do not need to control their emotions at all. Moreover, a regression analysis revealed that the subscale social isolation of the SVF120 was the strongest predictor for poor emotion regulation abilities (β = −0.467). This is especially intriguing when we consider that the s-allele has been linked to depression in older individuals, demonstrating how this genotype group prefers to focus on negative occurrences [40]. Another fascinating study [41] discovered that genetic variation in the 5HTTLPR influences memory and prefrontal cortex function in older persons. Another study [42] found that s-allele carriers have greater cortisol levels (a measure of elevated stress) and worse memory performance. This discovery has been interpreted as an increased sensitivity for s-allele carriers to dysregulation of the hypothalamic–pituitary–adrenal (HPA) axis, resulting in a decrease in hippocampus volume and memory [43]. This evidence is also coherent with the Strength and Vulnerability Integration Theory [44], which understands age-related vulnerabilities as physiological, provoking lower subjective well-being as well as less emphasis on positive information in the elderly.

### 3.4. CB1

Recently, researchers have begun to look at the role of the endocannabinoid system (EC) in cognitive-affective flexibility, which is dependent on working memory processes such as goal maintenance, inhibition, shifting, and updating. The endocannabinoid system is also linked to stress release, and CB1 receptors in the prefrontal cortex may influence anxiety, chronic stress, motivation, and social functioning in general [45]. CB1 receptors are required for the extinction of conditioned fear associations, implying that this receptor plays an important role in neuronal emotional learning and memory, as evidenced by fMRI studies on the cognitive control processes regulating fear extinction, with a focus on the role of the prefrontal cortex. The rs2180619 single-nucleotide cannabinoid receptor type 1 polymorphism (CB1 receptor) in the q14–ql5 region of chromosome 6 was the focus of research on the endocannabinoid system. By interfering with the release of other neurotransmitters, this receptor modulates the attenuation of synaptic transmission and psychoactivity. CB1 prevents the central nervous system from being overstimulated or inhibited by other neurotransmitters in this way. At least one copy of the minor “A” allele of the exonic rs1049353 polymorphism has been postulated as a protective factor against depression following stressful events, whereas homozygous bearers of the main G allele have been found to be at increased risk of antidepressant treatment resistance. In AA/AG genotype carriers, CB1 receptor activity increases emotion regulation strategies in facing stress, as well as consolidating emotional events into autobiographical memory [46]. Interestingly, the combination of CB1 and ADRA2B polymorphism has been related to improved working memory and positivity effect bias [47]. In particular, older adults with both ADRA2B and CB1 variants among 207 subjects (56 double-deletion carriers, 116 single-deletion carriers, and 35 non-deletion carriers) not only remembered more words in a working memory task (F(2, 169) = 5.790, *p* < 0.01, ɳp2 = 0.06), but they remembered more positively valenced words (F(2, 338) = 38.883, *p* < 0.001, ɳp2 = 0.19), showing a positivity effect bias. Indeed, CB1 receptors play a role in mood regulation and depression onset (e.g., CB1 receptor deficiency or antagonism can result in increased depressed behaviors) [48].

### 3.5. TOMM40

Mitochondrial activity in the brain is pivotal for neural plasticity, cognition, and many neural activities [49], whereas mitochondrial dysfunction occurs in a wide range of illnesses, including psychiatric and neurodegenerative disorders [50]. A novel gene polymorphism has been recently focused upon in the research on mitochondrial functioning (i.e., the gene encoding translocase of the outer mitochondrial membrane 40 (TOMM40), which is a protein importer). The TOM complex, of which TOM40 is the major pore, is responsible for importing most mitochondrial proteins from the cytoplasm into the mitochondria. Evidence [51,52] suggests that the length of the deoxythymidine homopolymer (poly-T) at rs10524523 (“523”) within intron 6 of the TOMM40 gene, which is typically classified as short (S) (14–20 repeats), long (L) (21–29 repeats), or very long (VL) (>29 repeats), is linked to differences in levels of risk for Alzheimer’s disease. The VL and L variants, for instance, have been linked to a greater risk of early onset of cognitive decline, whereas the S variant has been linked to improved memory and executive functioning [51]. The effect of TOMM40 on cognition has been found with both verbal and visuo-spatial stimuli, which is particularly notable [51]. To summarize, research is beginning to uncover the role of TOMM40 in several cognitive areas. The reasoning for this is that the TOMM40 gene is located near and in linkage disequilibrium with the apolipoprotein E gene (APOE), and hence it may mimic cognitive deficits observed in moderate cognitive impairment and Alzheimer’s disease. Some authors [53] have recently proposed a strong link between TOMM40 and high-likelihood or advanced AD. This might explain several discrepancies in TOMM40 research (e.g., conflicting data on the connection between TOMM40 and clinically diagnosed AD), as well as its role in the etiology of neurofibrillary tangles. The interplay between TOMM40 and APOE, on the other hand, is still open for debate, as TOMM40 may have effects that are independent of APOE [53].

TOMM40 rs2075650 (intron 2, chromosome 19q13.32, “650”), a single-nucleotide polymorphism (SNP), has been linked to cognitive and affective dysfunctions in addition to TOMM40 “523”. In particular, a study [54] involving emotionally charged material (an emotional word recall task with an immediate and delayed test) revealed that carriers of the minor (G) allele of TOMM40 have less positive memory bias (as measured by the number of positive intrusions on delayed recall) than AA carriers. These findings show how mitochondrial activity may play a role in the development of emotional biases. Given the well-known decreased efficiency of mitochondrial brain metabolisms in pathological aging [55], we recommend that future approaches to the investigation of positive effects on older individuals’ memory should include mitochondrial functioning. Most notably, the fact that dopaminergic neurons have a special sensitivity or vulnerability to mitochondrial stress supports the idea that mitochondrial dysfunction may be involved in modulating the direction of emotional biases [56]. Dopamine is very unstable, resulting in lower amounts of cellular reactive oxygen species (ROS), which is harmful to mitochondrial brain motility and survival. As a result, we believe TOMM40 and dopamine may interact to cause diverse patterns of emotional biases in the aging brain (see Figure 1 for a schematic summary of candidate genes involved in the generation of positivity effects in aging). The main features of the candidate genes in this review are listed in Table 1.

## 4. Discussion

### 4.1. A Positive Memory-Based Genetic Profile for Adaptation to the Space Environment

Positivity effects in memory are considered an index of well-being among healthy older adults [6]. Here, we review recent evidence that indicates a potential involvement of different genetic polymorphisms in driving the positivity effect in memory in line with a control-based account of the positivity effect that posits that regulating emotions requires cognitive control mechanisms to selectively attend and remember positive information. In particular, monoamines such as noradrenaline, dopamine, and serotonin play a crucial role in the regulation of emotions toward the positive pole. Our review identified a gene profile (ADRA2B plus CB1 deletions carrier, COMT Val 158 allele carrier, and L’L’ allele carrier of the 5HTTP gene) that may be linked to better emotion regulation abilities (in terms of favoring positive over negative information). In addition, dopamine metabolism seems to be crucial and is particularly sensitive to changes in mitochondrial functioning in the aging brain. In particular, AA carriers of the TOMM40 gene show a positivity bias in memory. Although this brief review draws mainly on few neurotransmitters, research has investigated the effects of interactions between different systems, showing mutual influence and modulation among these and other polymorphisms in aging [28]. It is, therefore, reasonable to suppose that all contribute to shaping valence effects in the aging brain. Recent progress in molecular genetics is introducing a new and more complete view of interpreting behavioral data. Understanding genes and their polymorphisms is relevant to the development of new a theoretical framework about the involvement of cell functioning in shaping memory. Hence, cell metabolism and particularly mitochondrial efficiency may be involved in shaping a valence bias in memory. In fact, according to results from mice studies [57,58,59,60,61,62,63,64,65], spaceflight stressors affect key regulators of brain neuroplasticity, including the neurotransmitters 5-HT and DA, as well as the neurotrophic factors CDNF and GDNF. In addition, genes associated with 5-HT (5-HT2A receptor and MAO A) and, in particular, DA (TH, MAO A, COMT, D1 receptor, CDNF, and GDNF) are among the main neurogenes at risk. A recent review [66] summarized the most relevant detrimental effects of long-term space missions on human biology, including oxidative stress [67], DNA damage [68,69], mitochondrial dysregulation [70], epigenetic changes [71], telomere length alterations [68,69], and microbiome changes [72]. In the NASA Twins Study [73], telomere elongation, which has been linked to Alzheimer’s disease [74], was found in Scott Kelly during his permanence on the ISS compared with his preflight, postflight, and terrestrial twin’s measurements. Scott’s telomere length, however, rapidly decreased after returning to Earth in just 48 h before gradually stabilizing at preflight levels over the course of the following 6 months. This finding may indicate that space-related detrimental effects may be transient.

**Figure 2:**
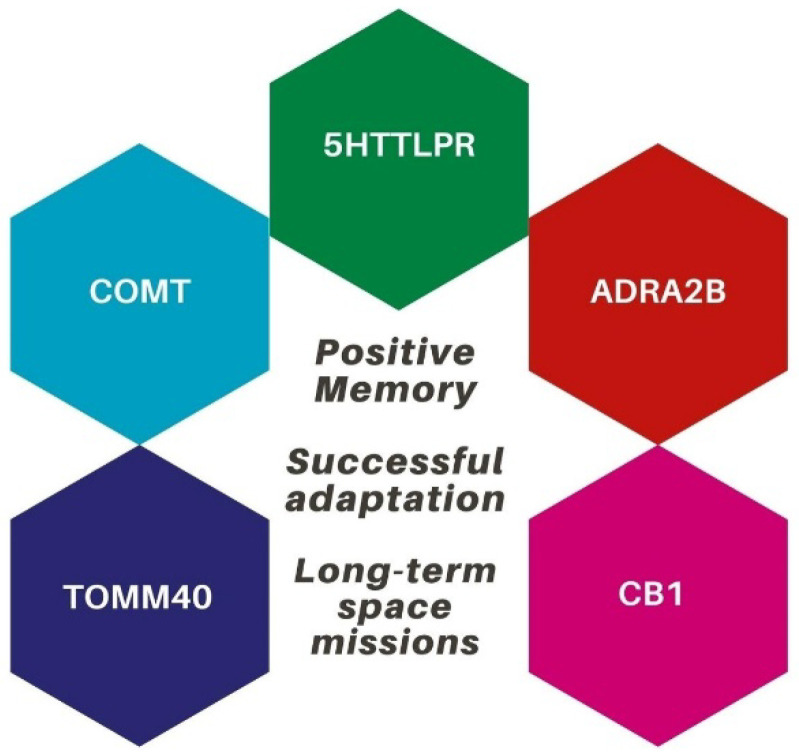
The genetic profile that may be involved in successful adaptation during space missions.

To summarize, the study of positive memory and adaptation’s genetics and epigenetics may have important implications in space sciences. Understanding the biological variables that underpin affective health may improve the chances of successful adaptation to extreme environments as well as to long-duration space missions (see Figure 2).

### 4.2. Hampering the Expression of the Positive Memory-Based Genetic Profile: The Case of Ionizing Radiations

Since affective health is rooted in underlying psychological, neurobiological, and physiological processes, other stressors that impact effective functioning may affect gene expression for positive memories. For instance, ionizing radiations (IR) are regarded as the most significant stressors for astronauts’ health during long-term missions [75], and they may influence positive memory-related gene expression. Despite the magnetic field of the Earth partially shielding astronauts from IR exposure in low-Earth orbit (LEO) spaceflights, on the ISS, astronauts are exposed to an average dose of 100–200 mSv/year [76], while on Earth, the radiation limit for health is 50 mSv/year [77]. IR will be a priority in deep space missions, considering that annual radiation doses for interplanetary expeditions will be around 350 mSv/year [78]. While research on MG on Earth uses established paradigms (HDBR and parabolic flights) to study its effects on humans, research on IR effects only uses animal models and cells in vitro or in vivo for obvious ethical reasons. Additionally, studies on space stressors are increasingly focusing on how different stressors interact with one another, particularly MG and IR. As described in the next paragraphs, ground-based research using animal models [79] shows that IRs have negative effects on the body, specifically the skeletal system, visual system, cardiovascular system, stem cells, and central nervous system (CNS), as well as negative effects on a variety of cognitive abilities, including memory. These findings indicate that our assumption about the importance of detecting a positive memory-based genetic profile to adapt to the space environment must take into account how IR may impact, alone or in combination with MG, a series of physiological and psychological mechanisms that may render the positivity bias less likely to occur during a mission.

For example, studies on mice have demonstrated that exposure to various radiations (protons and iron and carbon ions) damaged the skeletal system [80] even at low doses (such as 1–2 Gy of ^137^Cs) [81], provoking acute bone loss, probably due to decreased osteoblast activity and increased osteoclast activity. However, the effect of radiation on bones is still under investigation. In fact, in a study on skeletally mature mice (16 weeks of age) exposed to 0.5 Gy of ^56^Fe, some positive effects were documented [82]. Aside from IR, MG can be harmful to the skeletal system for many reasons (e.g., mechanical loading, altered calcium homeostasis, decreased hematopoiesis, and altered metabolism) [83]. While we do not have information on the long-term effects, the combined effects of IR and MG may be additive and primarily affect the structural aspect of the skeleton if not even impair the skeleton at the cellular level while leaving the biomechanical properties unaffected [84]. In addition, IR has been shown to negatively affect the visual system, particularly the retina and retinal vasculature, at high levels [84]. In ground-based studies on mice, low radiation doses resulted in a dose-dependent rise in retinal apoptosis [85]. Additionally, two years after proton irradiation, the loss of endothelial cells and microvessel lengths persisted, as well as oxidative stress and apoptosis in the retina [86]. The eyes are also adversely affected by microgravity due to altered tissue perfusion and the cephalic displacement of body fluids during parabolic flights (but not during HDBR) [85,87]. In long-duration missions, astronauts might experience an increase in intraocular pressure, as well as morphological changes in the optic nerve and its surrounding tissues [88]. The combined impact of radiation and microgravity on the visual system has not been fully investigated. In a study on mice [89], it was shown that hypogravity combined with low doses of proton irradiation (0.5 Gy) might compromise the survival of retinal endothelial cells. Moreover, IR seems to affect performance in multiple cognitive tasks and psychomotor vigilance in rats, provoking deficits in accuracy, impulsivity, and attention [84]. Managing the impact of IR on the CNS and cognitive performance should be a priority in long-term missions, considering that about 80% of flight accidents are caused by attention deficits [84]. MG, in addition, is a powerful CNS stressor, as it provokes changes to the brain’s structure (e.g., the rotation of the cerebral aqueduct) and a shift in cephalic fluid [90], and it affects sensorimotor and vestibular aspects [91]. Although the effects of MG on cognition appear to be transient and disappear soon after returning to Earth, at the cellular level, it might cause persistent changes in mitochondrial function and lipid metabolism in human oligodendrocytes, which are crucial for providing metabolic support to neurons [92]. The combined impact of IR and MG on the CNS and cognitive function is still poorly understood, and findings are inconsistent. Unusual exploratory behaviors or a high propensity for risk were discovered in mice exposed to hindlimb unloading (HLU), which did not worsen when in combination with low IR exposure [93].

Even at low doses, IR may have negative effects on the cardiovascular system [94,95]. Heavy ions (the so-called HZE particles) may have detrimental effects on cellular physiology, altering genes and upsetting redox metabolism, which leads to more oxidative stress, which in turn may deteriorate cardiovascular health. Vascular endothelial cells have been shown to be particularly sensitive to the effects of radiation [96]. Although a longitudinal study by NASA found that short-duration spaceflight does not increase the risk of adverse cardiac events [97], MG may be harmful to the cardiovascular system by lowering the cardiac workload, which leads to cardiac adaptation and uniform blood pressure [98]. It is unclear how MG and IR together will affect the cardiovascular system in long-duration missions. In a recent animal study [99], the combined effect of weightlessness simulated with HLU and space radiation with ^56^Fe ions was shown to impair endothelium-dependent vasodilation in the resistance arteries of the mouse gastrocnemius muscle immediately after treatments ended. In another study [100], however, this impairment in mice disappeared after 6–7 months, which is equivalent to 18–20 years in humans. Additionally, adverse effects of IR (in particular galactic cosmic rays or solar energy particles) have been discovered in stem cells, specifically in primitive and differentiated hematopoietic cell lines [84]. In particular, T-lymphocytes and B-lymphocytes have been shown to be radiosensitive [101], and this might indicate that IR could impair immune effectiveness. MG has similarly been shown to produce tissue degeneration and deficits in tissue regenerative health in mammalian stem cell-based processes, including hematopoiesis [102]. Several effects have been observed in some cell culture studies carried out in space, including the inhibition of osteoblast differentiation [103], reduction in osteoblast number [104], atrophy of skeletal muscle cells [105], impaired activation of immune cells and consequently the immune system [106], abnormal chondrocyte formation [107], and the collapse of the cytoskeleton in T-lymphoblastoid cells [108].

Finally, with regard to neurotransmission, acute IR seems to alter the neurotransmitter levels in the cerebral and cerebellar cortex in rats’ brains [109], in addition to microgravity being shown to cause reduced movements and lower expression levels of endogenous dopamine and COMT-4 due to dopamine-mediated functional impairments [110]. Interestingly, the injection of exogenous dopamine reversed the movement defects, as well as increased physical contact, improved COMT-4 and dopamine levels. If the findings are transferable to humans, manipulating the external environment might prevent muscle changes and represent a countermeasure for long-duration missions. In a study on rats [111], the combined effect of simulated hypogravity with HLU and IR with gamma rays and ^12^C^+6^ appear to increase noradrenergic neurotransmission in the prefrontal cortex and reduce serotonergic neurotransmission in the prefrontal cortex and amygdala. A study on humans found that both real (on the ISS) and simulated MG (parabolic flight) were linked to a significant increase in endocannabinoids (EC) in the blood, which gradually returned to baseline levels once back on the ground [112]. Enhanced EC signaling, according to the authors, may be necessary for adaptation and stress tolerance.

The above-mentioned literature indicates that, although we can identify a series of commonalities between the way healthy older adults cope with later life and astronauts with MG, the biological, genetic, and epigenetic perspectives on the “positivity effect” and the mechanisms involved in adaptation to the space environment deserve further investigation due to the complexity of space-related stress effects on the human body and mind. Nevertheless, we believe that this area of research is crucial in long-duration missions in order to develop efficient countermeasures to improve the affective health of astronauts. A neuro-bio-behavioral paradigm might include the astronaut selection program based on the neurogenetic “right stuff” profile, which may help detect some individuals less prone to affective disorders (a crucial aspect for long-term versus short-term missions), the biomarker-based health monitoring of in-mission astronauts, and the development of tailored countermeasures, as well as personalized medical therapies.

## Figures and Tables

**Table 1:** Main features of candidate gene carriers in relation to positive memory.

Candidate Genes	Main Role	Relationships with Positive Memory
**ADRA2B** **(209 bp/200 bp)**	Regulation of neurotransmitter release from sympathetic nerves and adrenergic neurons in the CNS	Deletion (200 bp) carriers show enhanced emotional stimuli processing and memory
		Deletion carriers have higher cognitive–affective flexibility compared with non-carriers
		Deletion carriers preserve emotional faces’ recognition abilities under acute stress conditions
**COMT** **(Val158/Met158)**	Metabolism of catecholamines and l-dopa	Met158 polymorphism is linked to increased neural activation for negative stimuli Met158 allele carriers have reduced performance in emotion-related tasks
		Met carriers have better WM performance, while Val carriers are better in processing negative stimuli
**5HTTLPR** **(S/S; L/L; S/L genotypes)**	The serotonin transporter (5-HTT) gene mediates the serotonin reuptake from the intersynaptic space	S-allele carriers show decreased left fusiform gyrus activation to positive stimuli
		S-allele carriers experience greater emotional responses to negative stimuli than l-carriers
		L-allele carriers are more efficient in emotion regulation than s-carriers
**CB1** **(AA/AG/GG genotypes)**	Modulate neurotransmission by inhibiting presynaptic Ca	Carriers of at least one copy of the minor A allele show improved consolidation for emotional events into autobiographical memory than GG genotype’s subjects
		Carriers of the combination of CB1 and ADRA2B polymorphisms show enhanced memory and a positive effect bias
		One copy of the minor A allele could be a protective factor against depression after stressful events
**TOMM40** **(intron 6: S/L/VL;** **intron 2: AA/GA/GG)**	Allows protein import from the cytoplasm into mitochondria	S variant of intron6 is associated with enhanced memory performance and executive functions
		AA genotype shows a positive memory bias compared with G-allele carriers
